# Influenza A virus targets a cGAS-independent STING pathway that controls enveloped RNA viruses

**DOI:** 10.1038/ncomms10680

**Published:** 2016-02-19

**Authors:** Christian K. Holm, Stine H. Rahbek, Hans Henrik Gad, Rasmus O. Bak, Martin R. Jakobsen, Zhaozaho Jiang, Anne Louise Hansen, Simon K. Jensen, Chenglong Sun, Martin K. Thomsen, Anders Laustsen, Camilla G. Nielsen, Kasper Severinsen, Yingluo Xiong, Dara L. Burdette, Veit Hornung, Robert Jan Lebbink, Mogens Duch, Katherine A. Fitzgerald, Shervin Bahrami, Jakob Giehm Mikkelsen, Rune Hartmann, Søren R. Paludan

**Affiliations:** 1 Department of Biomedicine, Aarhus University, Aarhus 8000, Denmark; 2 Aarhus Research Center for Innate Immunity, Aarhus University, Aarhus 8000, Denmark; 3 Department of Molecular Biology and Genetics, Aarhus University, Aarhus 8000, Denmark; 4 Division of Infectious Diseases and Immunology, Department of Medicine, University of Massachusetts, Amherst, Massachusetts M01003, USA; 5 Psychiatric Department, Aarhus University Hospital, Aarhus 8000, Denmark; 6 Department of Immunology, School of Basic Medical Sciences, Fudan University, Shanghai 200433, China; 7 Department of Molecular and Cellular Biology, University of California, Berkeley, California 94720, USA; 8 Institute of Molecular Medicine, University Hospital, University of Bonn, 53127 Bonn, Germany; 9 Department of Medical Microbiology, University Medical Center, Utrecht 3508GA, The Netherlands; 10 Skauvaccines, Aarhus Nord 8200, Denmark

## Abstract

Stimulator of interferon genes (STING) is known be involved in control of DNA viruses but has an unexplored role in control of RNA viruses. During infection with DNA viruses STING is activated downstream of cGAMP synthase (cGAS) to induce type I interferon. Here we identify a STING-dependent, cGAS-independent pathway important for full interferon production and antiviral control of enveloped RNA viruses, including influenza A virus (IAV). Further, IAV interacts with STING through its conserved hemagglutinin fusion peptide (FP). Interestingly, FP antagonizes interferon production induced by membrane fusion or IAV but not by cGAMP or DNA. Similar to the enveloped RNA viruses, membrane fusion stimulates interferon production in a STING-dependent but cGAS-independent manner. Abolishment of this pathway led to reduced interferon production and impaired control of enveloped RNA viruses. Thus, enveloped RNA viruses stimulate a cGAS-independent STING pathway, which is targeted by IAV.

## Abstract (web summary)

Stimulator of interferon genes (STING) is known to be involved in defence against DNA viruses, but its role in the control of RNA viruses remains poorly explored. Here the authors show that STING participates in an innate immune response to RNA virus infection in a cGAS-independent manner.

## Graphical abstract

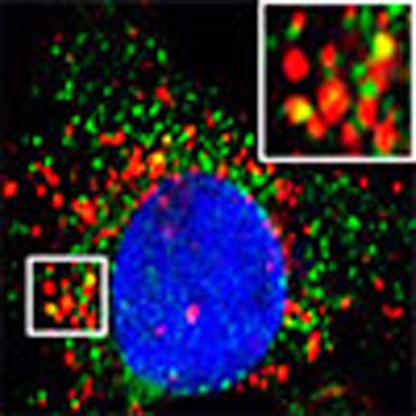

Cells respond to infections and alterations in cellular homoeostasis by stimulation of repair, death and defence mechanisms^1^. The innate immune system utilizes pattern-recognition receptors at cell surfaces, in endosomal compartments, and in the cytoplasm to detect immunostimulatory molecules and induce antimicrobial activities^2^. In the cytoplasm, RNA species are detected by RIG-I-like receptors (RLRs), and cytosolic DNA is sensed by cyclic GMP-AMP (cGAMP) synthase (cGAS)^3^,^4^. While signalling stimulated by RNA proceeds through a pathway dependent on the adaptor mitochondrial antiviral-signalling (MAVS) protein, DNA-stimulated signalling relies on stimulator of interferon genes (STING)^5^. Upon DNA recognition, cGAS produces 2'3' cGAMP, which binds STING to induce a conformational change. This allows activation of the TANK-binding kinase 1 (TBK1) (refs 6, 7), which phosphorylates and activates the transcription factor IRF3 leading to expression of type I interferons (interferon, interferon-α/-β)^5^,^6^,^7^,^8^.

STING was first identified as being important in innate immune responses to RNA viruses^9^. STING-deficient murine embryonal fibroblasts (MEFs) exhibit increased viral growth when infected with vesicular stomatitis virus (VSV) and decreased interferon-β production in response to Sendai virus (SeV). The importance of STING in the innate immune defence against RNA viruses was further supported by the identification of RNA viruses that antagonize STING-dependent signalling^10^,^11^,^12^. In contrast to the well-documented role for STING in innate responses to DNA viruses, the basis for the involvement of STING in controlling RNA viruses has not been identified. Here we describe a cGAS-independent STING pathway triggered by enveloped RNA viruses to evoke innate immune responses and demonstrate that this pathway is targeted by influenza A virus (IAV).

## Results

### A role for STING but not cGAS in responses to RNA viruses

**Figure 1:**
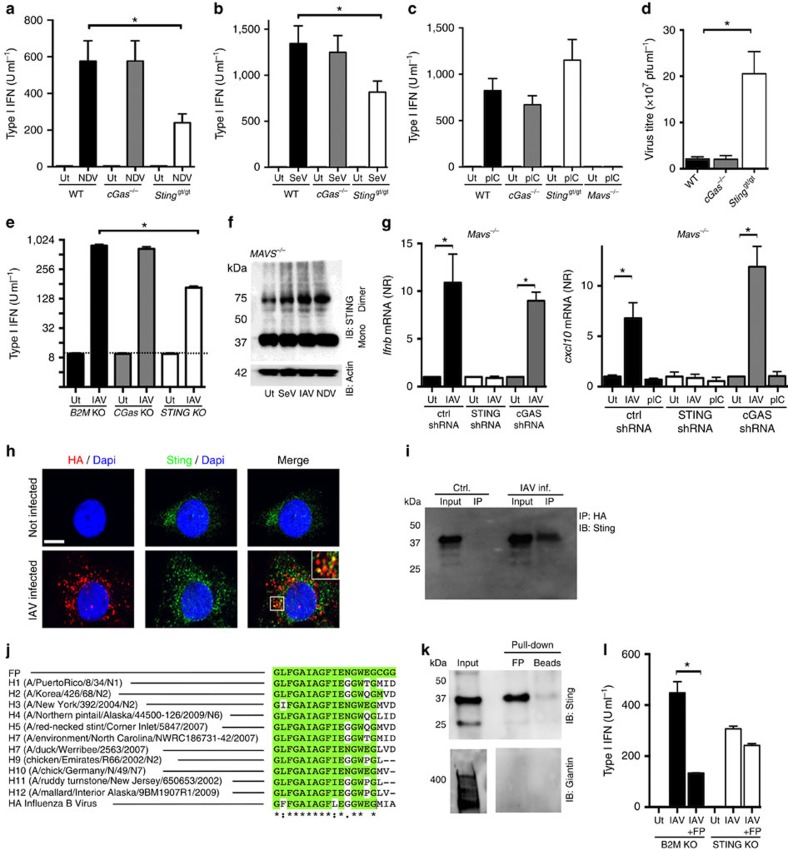
A role for STING but not cGAS in innate antiviral responses against RNA viruses. (**a**–**d**) MEFs from C57BL/6, cGAS^−/−^ and STING^gt/gt^ mice were infected with (**a**) NDV (MOI 1), (**b**) SeV (MOI 0.1), (**d**) VSV (MOI 0.01) or (**c**) transfected with poly(I:C; 2 μg ml^−1^). Supernatants were isolated 16–20 h later and levels of type I interferon (IFN) and viral titre were quantified by interferon bioassay and end point titration, respectively. Data are presented as means of four biological replicates +/− s.d. (**e**) THP1 cells deficient in β2 microglobulin (*B2M KO*), cGAS (*cGAS KO*), or STING (*STING KO*) were infected with IAV (MOI 10). Supernatants were harvested 16 h later and analysed for interferon-α/-β bioactivity. Data are presented as means of four biological replicated ±s.d. (**f**) MAVS-deficient THP1 cells were infected with SeV, IAV or NDV for 8 h, and lysates were analysed for levels of STING dimers by western blotting using non-reduced SDS-PAGE. (**g**) MAVS-deficient THP1 cells transduced with Control, cGAS or STING shRNA were infected with IAV and RNA collected at 5 h and 20 h post infection were analysed for levels of interferon-β and CXCL10 mRNA, respectively, by RT–qPCR. (**h**) THP1 cells were infected with IAV for 60 min and stained for hemagglutinin (HA; red), STING (green) and DAPI (blue) and analysed by confocal microscopy. (**i**) Immunoprecipitation of hemagglutinin was performed on lysates from THP1 cells infected with IAV for 1 h. The precipitate was immunoblotted for STING. (**j**) N-terminal HA2 sequences from IAV strains and influenza B Virus. (**k**) THP1 cell lysates were mixed with recombinant biotin-labelled FP. Eluate from streptavidin beads was analysed by immunoblotting. (**l**) THP1 cells deficient in *B2M KO* and *STING KO* THP1 cells were pretreated with 5 μM of FP and infected with IAV. Supernatants were collected 16 h later and analysed for interferon-α/-β bioactivity. All experiments were performed at least three times with similar results. *P<0.05 by Mann–Whitney compared-ranks test.

RNA viruses induce type I interferon expression in a manner dependent on the RLR-MAVS pathway^13^. However, STING is also necessary for full responsiveness to—and control of—RNA viruses^9^. Work by us and others has recently proposed alternative and DNA-independent means for activating STING-dependent innate antiviral immune responses^14^,^15^. In line with this, RNA viruses have been demonstrated to target the STING pathway^10^,^11^,^12^. In order to evaluate the role of STING in the stimulation of interferon production by RNA viruses, and to explore the potential involvement of cGAS, we infected wild-type (WT), STING- and cGAS-deficient MEFs with the two paramyxoviruses Newcastle disease virus (NDV) and SeV and measured the accumulation of type I interferon in the culture supernatant. Interestingly, lack of STING, but not cGAS, significantly reduced the production of type I interferons by cells infected with these viruses (Fig. 1a,b, Supplementary Fig. 1a). In contrast to this, the stimulation of interferon expression by synthetic RNA (poly(I:C)) was not affected in cells lacking STING or cGAS but was completely abolished in MAVS-deficient cells (Fig. 1c and Supplementary Fig. 1b). To examine whether STING but not cGAS contributed to control of RNA viruses, we infected MEFs from WT, cGAS- and STING-deficient mice with the rhabdovirus VSV and measured accumulation of progeny virus in the supernatant. Consistent with the data on interferon production by RNA viruses, lack of STING but not cGAS led to elevated VSV replication (Fig. 1d).

To evaluate the role of the cGAS-independent STING pathway in stimulation of interferon production by a human pathogenic RNA virus, we tested IAV. Since, IAV did not induce detectable type I interferon production in MEFs we used the human monocyte/macrophage-like cell line THP1. CRISPR/Cas9 technology was used to generate cells deficient for cGAS and STING or β2microglobulin (B2M) as control. The deletion of cGAS and STING was confirmed by western blotting (Supplementary Fig. 1c). As expected, the loss of cGAS or STING abolished the ability to produce type I interferon in response to transfection with DNA, whereas dsRNA-induced type I interferon production was unaffected (Supplementary Fig. 1d). The cells were infected with IAV, and accumulation of interferon in the supernatant was measured after 20 h. Similar to what was observed with NDV and SeV in MEFs, we observed that IAV-induced interferon production was reduced in cells lacking STING expression, but not in cGAS-deficient THP1 cells (Fig. 1e).

It is well-established that RNA viruses induce type I interferon in a manner dependent on the RLR-MAVS pathway^13^. Consistent with this, the majority of the interferon responses evoked by infection with NDV or SeV were dependent on MAVS (Supplementary Fig. 2a,b). However, we observed a significant residual response in MAVS-deficient cells infected with RNA viruses, which we did not observe when transfecting with synthetic RNA (Supplementary Fig. 2a,b and Fig. 1c). The MAVS-independent nature of interferon responses was also observed for IAV, when measuring the ISG CXCL10 in BMDCs infected with high multiplicity of infection (MOI) (Supplementary Fig. 2c). To further explore whether RNA viruses induced a MAVS-independent but STING-dependent pathway, we examined for the ability of SeV, NDV and IAV to stimulate STING dimer formation in THP1 cells lacking MAVS, and therefore unable to respond to RNA (Supplementary Fig. 1a). Interestingly, infection with either of the three RNA viruses induced elevated levels of STING dimers in the lysates (Fig. 1f), and consistent with this, the residual induction of interferon-β and CXCL10 expression by IAV in MAVS-deficient cells was ablated when STING but not cGAS was knocked down by shRNA (Fig. 1g and Supplementary Fig. 2d). Poly(I:C) did not stimulate CXCL10 expression in MAVS-deficient cells (Fig. 1g). These data suggests a role for STING in evoking innate immune responses to RNA viruses through a non-canonical pathway.

Identification of viral evasion strategies provides strong evidence for an important role of an immune pathway in antiviral defence^16^. For instance, IAV seeks to evade the RIG-I pathway by the non-structural protein 1, which targets the E3 ubiquitin ligase TRIM25 responsible for RIG-I CARD ubiquitination^17^. Interestingly, co-staining of cells with antibodies against the IAV surface protein hemagglutinin and STING revealed that hemagglutinin co-localized with a small pool of cellular STING (Fig. 1h). The observed specks staining positive for both STING and hemagglutinin, was observed in the majority of cells with positive hemagglutinin staining. Among the STING-hemagglutinin double-positive specks, many were also positive for the early endosome antigen 1 (Supplementary Fig. 3), potentially suggesting the co-localization to occur in early endosomal compartments. Moreover, STING co-immunoprecipitated with hemagglutinin in lysates from infected cells (Fig. 1i). Hemagglutinin is involved in viral entry, and the N-terminal region of HA2, termed the fusion peptide (FP), penetrates the cellular membrane during fusion^18^. FP is highly conserved among IAV strains and also in influenza B virus^18^,^19^ (Fig. 1j), and was recently shown to have immunosuppressive properties in a monocytic cell line^20^. Remarkably, recombinant FP interacted readily with STING in cellular lysates but not with Giantin (Fig. 1k), which, similarly to activated STING, localizes to the Golgi.

To investigate if the interaction between STING and FP represented a strategy for IAV to evade STING-dependent induction of interferon production we pretreated THP-1-derived B2M and STING knock-out (KO) cells with FP before infection with IAV. Interestingly, pretreatment with FP led to a significantly decreased interferon-α/-β response in B2M KO cells but not in the STING-KO THP-1 cells (Fig. 1l). Collectively, these data demonstrate that a STING-dependent but cGAS-independent pathway is involved in stimulation of the interferon response to—and control of—RNA viruses, but not in the interferon response to synthetic RNA. Moreover, the IAV FP is able to directly interact with STING.

### IAV FP inhibits fusion-stimulated interferon production

**Figure 2:**
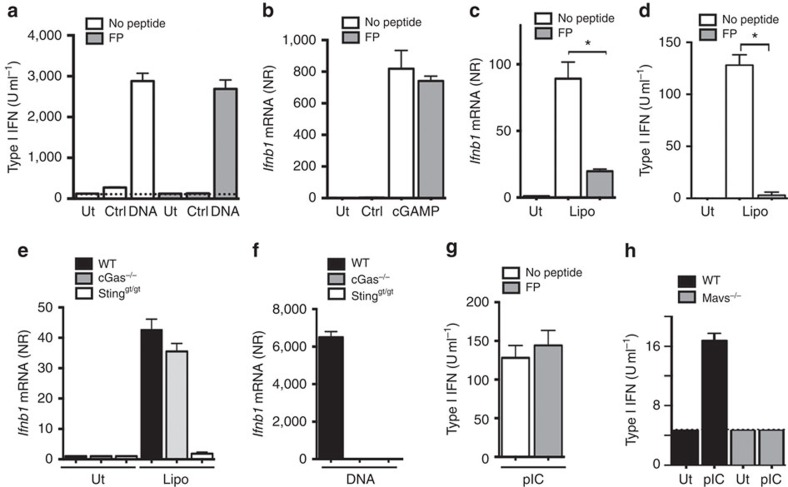
FP from IAV inhibits interferon production selectively in response to fusogenic liposomes. (**a**–**d**,**g**) BMDCs were pretreated with 5 μM of FP and stimulated with dsDNA (4 μg ml^−1^), cGAMP (10 nM), fusogenic liposomes (Lipo, 2 μg ml^−1^) or poly(I:C; 4 μg ml^−1^) as indicated. Supernatants and total RNA were collected 16 h post-stimulation and analysed for interferon-α/-β bioactivity or interferon-β mRNA (normalized to β-actin, NR). (**e**,**f**) BMDCs from C57BL/6, cGAS^−/−^ and Sting^gt/gt^ mice were treated with Lipo (2 μg ml^−1^) or dsDNA (4 μg ml^−1^) as indicated. Total RNA was collected 6 h later and analysed for or interferon-β mRNA (normalized to β-actin, NR). (**h**) BMDCs from C57BL/6 and MAVS^−/−^ mice were treated with poly(I:C; 4 μg ml^−1^). Supernatants were collected 16 h later and analysed for type I interferon (IFN) bioactivity. Data are presented as means of four biological replicates (±s.d.). *P<0.05 by Mann–Whitney compared-ranks test. NS, not significant. All panels represent data from one of at least two independent experiments.

We wanted to investigate whether FP affected known STING-dependent responses, including type I interferon production after stimulation with DNA, cGAMP or fusogenic liposomes^5^,^6^,^7^,^8^,^14^. When treating cells with recombinant FP there was no detectable effect on responses to DNA or cGAMP (Fig. 2a,b). By contrast, FP largely abrogated type I interferon production induced by fusogenic liposomes (Fig. 2c,d), without affecting fusion between liposomes and cells (Supplementary Fig. 4a). Furthermore, as we have also demonstrated previously^14^, fusogenic liposomes stimulated STING speck formation (Supplementary Fig. 4b), and as observed with IAV the production of type I interferon stimulated by fusogenic liposomes was STING-dependent but cGAS-independent (Fig. 2e,f). FP did not affect the MAVS-dependent response to synthetic RNA, as the response to transfection with poly(I:C) was unchanged (Fig. 2g,h). Moreover, by introducing a single amino-acid deletion to FP (FPΔI6) the ability to block type I interferon production was abrogated (Supplementary Fig. 4c). Thus, IAV FP specifically inhibits the cGAS-independent STING pathway induced by lipid membrane fusion but does not affect interferon induction by cytosolic delivery of synthetic DNA, cGAMP or RNA.

### FP inhibits STING activation in response to membrane fusion

**Figure 3:**
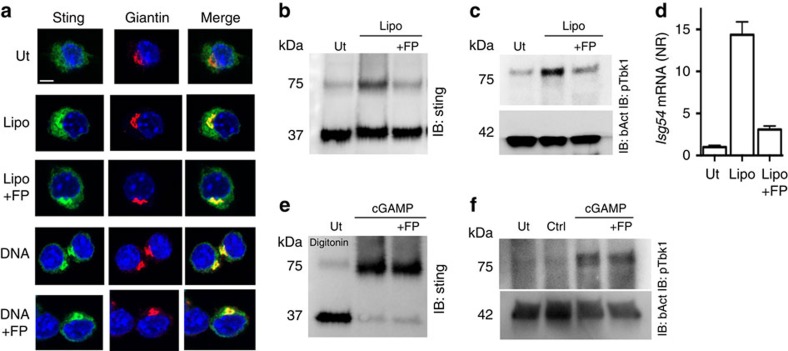
The fusion peptide from IAV hemagglutinin inhibits STING dimerization and TBK1 phosphorylation in response to membrane fusion. (**a**) iBMDMs expressing GFP-tagged STING were stimulated with dsDNA or with fusogenic liposomes (Lipo, 2 μg ml^−1^). Cells were pretreated with FP for 15 min or left untreated. After 1 h, cells were fixed and stained for giantin and with DAPI. White scale bar is 5 μm. (**b**–**f**) THP1 cells were pretreated with FP (10 μM) as indicated and stimulated for 3 h. Cell lysates were prepared and separated by SDS-PAGE under non-reducing (**b**,**e**) or reducing (**c**,**f**) conditions and immunoblotted for STING, pTBK1 or β-actin. All panels represent data from one of at least two independent experiments. (**d**) Total RNA was isolated 4 h after stimulation and levels of ISG54 mRNA were determined by RT–qPCR, normalized to β-actin and shown as means±s.e.m.

Next we wanted to identify the step in the fusion-activated signalling pathway that was targeted by FP. Stimulation through the cGAS-STING pathway is reported to initiate and depend on relocation of STING from the endoplasmic reticulum (ER) to the Golgi^5^. Similar to DNA stimulation, treatment with fusogenic liposomes triggered translocation of STING to the Golgi (Fig. 3a), and Brefeldin A inhibited interferon-β expression in response to either fusogenic liposomes or DNA (Supplementary Fig. 5a), thus supporting the importance of STING translocation from ER to the Golgi. Treatment with FP did not interfere with translocation of STING from ER to the Golgi (Fig. 3a). STING forms covalent homodimers in response to stimulation with cyclic di-nucleotides, and dimerization of STING is sufficient for induction of type I interferon^21^. When examining for STING dimer formation using non-reducing gels, we found that treatment with fusogenic liposomes induced the formation of a reducible STING dimer (Fig. 3b and Supplementary Fig. 6a). Interestingly, FP pretreatment abolished STING dimerization, subsequent TBK1 phosphorylation and IRF-dependent transcription in response to fusogenic liposomes (Fig. 3b–d) but not in response to cGAMP irrespective of delivery method (Fig. 3e,f and Supplementary Fig. 6b). These data demonstrate that stimulation of STING-dependent pathways results in formation of stable STING homodimers and phosphorylation of TBK1, and that the IAV FP specifically inhibits this in response to fusogenic liposomes.

### FP binds STING in the region of the dimerization interphase

**Figure 4:**
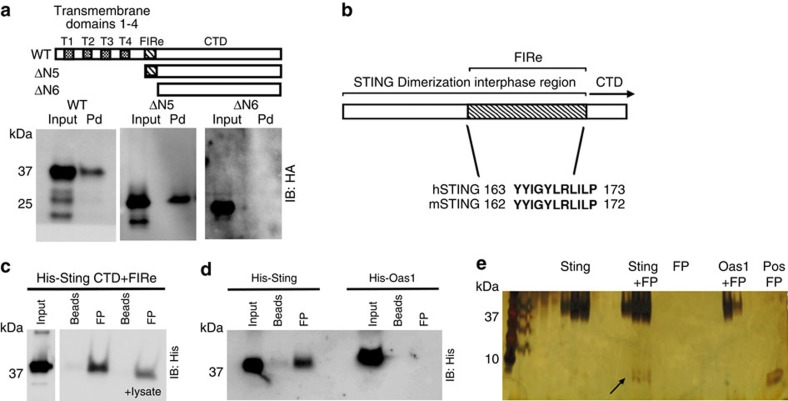
The IAV HA2 FP binds STING. (**a**) Hemagglutinin (HA)-tagged mSTING was expressed in HEK293T cells, lysates were mixed with biotinylated FP and precipitated with streptavidin beads. Co-precipitated STING was detected by immunoblotting. (**b**) Graphic presentation of the STING region encompassing the dimerization interphase and the FP-interacting region (FIRe). (**c**,**d**) His-tagged hSTING CTD+FIRe hSTING (**c**,**d**) or His-tagged pOAS1 (**d**) were mixed with biotinylated FP and precipitated using streptavidin beads. In **c** the cellular lysates from HEK293 cells were added as indicated. The precipitates were analysed for hSTING or pOAS1 by immunoblotting for His. (**e**) Solutions of hSTING CTD+IFRe alone, hSTING CTD+IFRe plus FP, FP alone and pOAS1 plus FP were subjected to analytical SEC. High molecular fractions were separated by SDS-PAGE and silver-stained together with a positive control for FP (Pos FP). All experiments were performed at least two times with similar results.

To identify the region of STING interacting with FP we expressed hemagglutinin-tagged mSTING mutants^22^ in HEK293T cells. Very little STING was detected in the reactions incubated with beads alone (Supplementary Fig. 6c). By contrast, FP-coupled beads efficiently precipitated WT and the ΔN5 (aa162-378) STING mutant but not the ΔN6 (aa173-378) STING mutant (Fig. 4a). Notably, the FPΔI6 mutant peptide failed to precipitate STING (Supplementary Fig. 4d). These data identify the residues 162–172, which are highly conserved between murine and human STING, as being important for the interaction between STING and FP (Fig. 4b). Moreover, the region contains residues involved in binding to cGAMP^6^,^7^ and overlaps with a region important for STING dimerization^4^. To investigate whether the interaction between FP and STING was direct, we expressed hSTING (C-terminal domain containing the FP-interacting region; CTD+FIRe) in *E. coli* and mixed purified hSTING with biotinylated FP. In support of a direct interaction, FP co-precipitated with STING but not 2′-5′-oligoadenylate synthetase 1 (OAS1), which was used as control (Fig. 4c,d). Addition of cellular lysate to the mix of recombinant STING and FP did not increase the interaction, suggesting no requirement for other cellular factors (Fig. 4c). Moreover, a direct interaction between STING and FP was confirmed by size exclusion chromatography (SEC) (Fig. 4e). Together, these data demonstrate that IAV hemagglutinin interacts directly with STING through the FP in a manner dependent on a specific and conserved region in STING.

### Sensing of fusion is central for full control of RNA viruses

**Figure 5:**
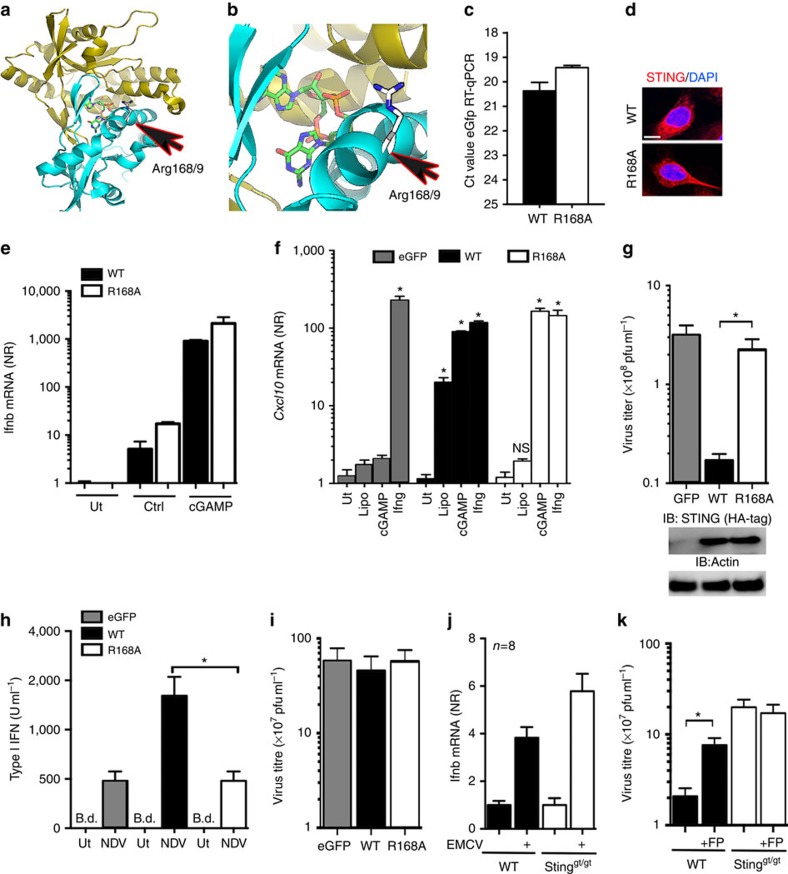
The fusion-activated STING pathway is essential for full innate control of RNA viruses. (**a**,**b**) Illustration of hSTING dimer (monomers shown in yellow and cyan) depicting the position of Arg169 relative to the position of cGAMP. (**c**–**j**) WT mSTING, R168A mSTING or eGFP were stably expressed in MEFs or BMDCs from *Tmem173*^gt/gt^ mice. Scale bar, 5 μm. (**c**) Transduction efficiency was assessed in BMDCs by RT–qPCR analysis of measuring eGFP transcripts. (**d**) Laser-scanning microscopy image of mSTING expression in WT and R168A STING MEFs. (**e**) WT and R168A STING MEFs were stimulated with cGAMP and analysed for interferon-α/-β by RT–qPCR. (**f**) BMDCs were stimulated with fusogenic liposomes (Lipo, 2 μg ml^−1^), cGAMP (10 nM) or interferon-γ (1 ng ml^−1^). After 6 h m*Cxcl10* mRNA was analysed by RT–qPCR. (**g**) MEFs expressing either WT or R168A mSTING were infected overnight with VSV (MOI 0.01). Virus production was determined by end point titration. The western blot below the graph show the levels of STING and β-actin in the cells used for experiments. (**h**) BMDCs were pretreated with FP where indicated and infected with NDV. Supernatants were collected after 20 h and analysed for interferon-α/-β bioactivity. (**i**,**j**) MEFs expressing either WT or R168A mSTING were infected overnight with EMCV (MOI 0.01). Virus production was determined by end point titration. WT or Sting^gt/gt^ cells infected with EMCV (MOI 0.1) for 6 h were lysed and total RNA was analysed for levels of interferon-β mRNA, normalized to β-actin and presented as normalized ratios relative to uninfected controls. (**k**) MEFs from C57BL/6 and STING^gt/gt^ mice were pretreated with 10 μM of FP and infected VSV (MOI 0.01). Supernatants were isolated 20 h later and viral titres were quantified by end point titration. The numerical data are presented as means of four biological replicates (±s.d.). *P<0.05 by Mann–Whitney compared-ranks test. IFN, interferon; HA, hemagglutinin. All graphs represent data from one of at least two independent experiments. b.d. indicates measurements below the detection limit.

Residues 162–172 are found in a central helix, which lines the cGAMP-binding pocket and participates in the formation of the STING dimer^4^. We noted that Arg168 (Arg169 in hSTING) is located outside the STING dimerization surface, is facing away from the cGAMP-binding pocket, is surface exposed and is thus unlikely to be important for protein folding (Fig. 5a,b). Nevertheless, Arg168 is highly conserved among vertebrates^23^. To determine whether Arg168 was involved in signalling in responses to lipid membrane fusion and infection with RNA viruses, we reconstituted BMDCs and MEFs from STING-deficient mice with WT STING, R168A STING or eGFP. Similar transduction efficiency was obtained between WT and R168A constructs, and we also observed comparable distribution of WT and R168A STING in the reconstituted cells (Fig. 5c,d). The expression of either WT or R168A STING restored the responsiveness to cGAMP (Fig. 5e,f). By contrast, expression of WT but not R168A restored responsiveness to fusogenic liposomes (Fig. 5f). The opposite was observed when we tested STING-deficient cells reconstituted with R231A (Supplementary Fig. 7), which like R232 in human STING is essential for responsiveness to cyclic-di-nucleotides^22^. Thus, the fusion-STING and the cGAS-cGAMP-STING pathways can be functionally separated by the two mutants tested, and the R168A mutant provided a tool that enabled us to functionally study the fusion-STING pathway. Interestingly, when infected with VSV, production of progeny virus was reduced ∼10-fold in cells expressing WT STING compared with R168A and eGFP-expressing cells (Fig. 5g). Moreover, type I interferon production in response to NDV was augmented by expression of WT but not R168A STING (Fig. 5h). VSV and NDV are both enveloped viruses. To determine whether R168A was also important for control of a naked RNA virus, we evaluated replication and interferon-β mRNA expression during infection with the picornavirus encephalomyocarditis virus in these cell lines. Interestingly, expression of neither WT nor R168A STING affected the replication of encephalomyocarditis virus (Fig. 5i), and the modest induction of interferon-β expression was independent of STING (Fig. 5j). Finally, we wanted to examine whether the IAV FP could antagonize the STING-dependent antiviral response against VSV. STING-deficient MEFs transduced with eGFP or WT STING were treated with vehicle or FP during infection. Interestingly, treatment with FP led to elevated viral replication in cells expressing STING but not in cells lacking STING expression (Fig. 5k). Collectively, these data show that sensing of lipid membrane fusion contributes to the antiviral response against enveloped RNA viruses and identifies the IAV FP as a viral strategy to counteract this defence mechanism.

## Discussion

The innate immune system is responsible for early protection against infections, and microorganisms have evolved mechanisms to evade innate immune responses to promote infection^16^. DNA viruses are detected by cGAS, which produces 2′3′ cGAMP to activate STING through direct binding resulting in stimulation of the TBK1-IRF3-interferon pathway^24^. For RNA viruses, it is known that RIG-I and MDA5 detect viral genomic material to stimulate signalling via MAVS to the TBK1-IRF3-interferon pathway^13^. However, after infections at higher MOI, residual RLR-MAVS-independent interferon production has been observed^25^,^26^,^27^. Moreover, full protection against RNA viruses also relies on STING^9^, and many RNA viruses have developed mechanisms to block STING-dependent signalling^10^,^11^,^12^. Despite this, there is no knowledge on the nature of STING-dependent pathway that is important for innate responses to RNA viruses^10^,^11^,^12^. Here we identify a STING-dependent but cGAS-independent pathway, which is stimulated by lipid membrane fusion, and is important for type I interferon expression in response to enveloped RNA viruses, including IAV. Furthermore, we demonstrate that the IAV HA2 FP targets this pathway through direct binding to STING and inhibition of STING dimerization and activation of TBK1 (Supplementary Fig. 8).

A key finding of the present article is that enveloped RNA viruses, including IAV, and fusogenic liposomes both stimulate type I interferon expression in a manner dependent on STING but not cGAS. In contrast, DNA stimulates the same response through a pathway dependent on both cGAS and STING. These data suggest that the process of virus-cell membrane fusion is responsible for stimulation of STING-dependent signalling by enveloped RNA viruses. We previously reported that herpes simplex virus-like particles induce low-grade interferon-β expression through fusion with the cell plasma membrane^14^, and the present data suggests that this mechanism is involved in mounting antiviral responses against enveloped but not naked RNA viruses. It still remains unresolved how the fusion of viral membranes with the cell is detected to initiate signalling.

A second conclusion from the present study is that STING-dependent signalling can be activated by additional mechanisms other than through binding to cGAMP. To this end, our identification of Arg168 (Arg169 in human STING) as being important for stimulation of interferon expression by liposomes but not cGAMP, demonstrates that activation by these two stimuli can be mechanistically separated. Importantly, the STING R168A mutant exhibited impaired ability to evoke full interferon and antiviral responses during infection with RNA viruses. Arg168 is located just outside the STING dimerization surface, is facing away from the cGAMP-binding pocket and is surface exposed^4^,^6^,^7^. Therefore, it is possible that this positively charged and highly conserved residue is involved in protein–protein interactions with yet unknown partner(s), which are recruited upon membrane fusion and are involved in stimulating STING-dependent signalling. With respect to the existence of mechanisms of STING activation independent of DNA stimulation and cGAMP engagement, patients with gain-of-functions mutations in STING and constitutive activation of the pathway were recently reported^28^.

Fusion-stimulated STING-dependent signalling was inhibited by the IAV FP. This suggests that the pathway is of importance for defence against IAV infections. hemagglutinin is essential for the fusion of IAV with the host cell, and FP is engaged in the membranous region during fusion^18^. We found that hemagglutinin did co-localize with a small pool of STING early during infection, and STING was precipitated with hemagglutinin in cell lysates from infected cells. The observation that most STING did not co-localize with hemagglutinin could suggest that the STING–hemagglutinin interaction is very transient in nature or that only the pool of STING engaged in sensing of fusion is targeted by hemagglutinin. Since STING needs to be activated to stimulate signalling, and the cGAS-STING pathway is not activated by RNA viruses, our data could suggest that hemagglutinin selectively targets the pool of STING recruited to endosomes, thus preventing fusion-stimulated signalling.

The finding of interaction between hemagglutinin and STING associate during infection was complemented with data demonstrating interaction between FP and STING. FP interacted directly with STING in the region between the residues 162 and 172 on mSTING and antagonized dimerization, thus preventing TBK1 phosphorylation and interferon expression. The HA2 FP is highly conserved between IAV strains^20^, and future work should seek to mechanistically dissect the action of FP in viral entry versus STING interaction. This could potentially allow studies with viruses able to enter into cells but unable to evade the STING pathway, and would also lead to better prediction of pathogenicity of emerging IAV strains. It is known that IAV targets the RIG-I pathway. The non-structural protein 1 interacts with RIG-I and antagonizes downstream signalling via MAVS by inhibiting the TRIM25 E3 ligases responsible for RIG-I CARD ubiquitination^17^. Thus, together with the data presented in this work, there is now evidence to support that IAV has evolved means to evade signalling through both MAVS- and STING-driven pathways.

Collectively, this work establishes a novel cGAS-independent mechanism of STING activation, which is involved in achieving full antiviral responses against enveloped RNA viruses. A key question now emerging is what might be the mechanisms governing the non-canonical mechanism of cGAS-independent STING activation.

## Methods

### Reagents and cells and viruses

All experiments with primary mouse cells are approved by the local Danish ethics committee for animal experiments (dyreforsøgstilsynet). Taqman assays for PCR were from Life Technologies. BMDCs were differentiated from bone marrow using GM-CSF (R&D systems) 7-day cultures. cGAMP (cyclic (guanosine-(2′-5′)-monophosphate-adenosine-(3′-5′)-monophosphate) was purchased from BioLog. Anti-Giantin was from Abcam. Antibodies for STING and pTBK1 immunoblotting were from Cell Signaling Technology. SDS-PAGE and protein transfer was performed using the Biorad Criterion system. Beads for precipitation studies were purchased from Life Technologies. Lipid blends containing DOTAP, DOPE and lissamine–rhodamine DOPE in the w/w/w ratio 1/1/0.1 were purchased from Avanti Polar Lipids and liposomes were prepared as described previously^14^. In brief, lipids were dissolved in chloroform in the indicated ratios. Chloroform was then evaporated under inert gas pressure leaving a thin lipid film. Liposomes were formed by hydrating the lipid film with phosphate-buffered saline. Uniform size of liposomes weas achieved by passing the hydrated lipids through a 0.2 μm pore filter using a mini-extruder purchased from Avanti Polar lipids. FP GLFGAIAGFIENGWEGC from IAV (FP)^20^. A tail of GGEKEKEK was added to the peptide to achieve solubility. Peptides were produced as covalent homodimers linked through sulfur bridges. Sequence of deletion peptide FPDI6 was GLFGAAGFIENGWEGC. Peptides were purchased from the company Schafer-N. STING-GFP expressing iBMDMs were kindly donated by Dr Melanie Brinkmann (Braunschweig, Germany). IAV was purchased from ATCC (H1N1, PR8). VSV was of the Indiana strain and NDV and SeV were kindly provided by professor Peter Palese^29^ and professor Illka Julkunen, respectively. BMDCs were prepared by 7-day culturing of bone marrow cells with GM-CSF and then collecting non-adherent cells. STING- and cGAS-deficient MEF were obtained from embryos of Golden ticket and cGAS^−/−^ mice at E13.5. Primary cells were transformed with lentivirus containing SV40 and positive cells were selected with puromycin. Transformed cells were re-infected with lentivirus containing lentiviral expression system for STING or mutant forms of STING. For lentiviral reconstitution of STING expression in BMDCs from STING-deficient mice, bone marrow was collected and placed in culture supplemented with GM-CSF. Next day lentivirus was added to the cultures. Cells were cultured with GM-CSF until day 7 whereupon the non-adherent cells were collected. For stimulation of cell cultures with IAV, PR8 strain was used at a MOI of 60 HAU per 10^6^ cells for 20 h.

### Knockout using the CRISPR-Cas9 system

We used the CRISPR/Cas9 system to generate B2M, cGAS and STING-KO THP1 cells. We used a lentiviral CRISPR/Cas9 vector^30^ encoding a codon-optimized nuclear-localized Cas9 gene N-terminally fused to PuroR via a T2A ribsome-skipping sequence. In addition, the vector contains a human U6 promoter driving expression of a guideRNA consisting of a gene-specific CRISPR RNA (crRNA) fused to the *trans*-activating crRNA (tracrRNA) and a terminator sequence. The gene-specific crRNA sequences cloned were: 5′-GAGTAGCGCGAGCACAGCTA-3′ for B2M, 5′-GAGCACACTCTCCGGTACC-3′ for STING and 5′-GACTCGGTGGGATCCATCG-3′ for cGAS. THP1 cells were transduced with CRISPR/Cas9 lentivirus, and selected with 2 μg ml^−1^ puromycin for 2 weeks. Cells were subsequently cloned by limiting dilution, and individual clones were subjected to western blotting to confirm absence of the targeted gene products. The MAVS-deficient THP-1-derived cells were generated as described previously^31^. In brief, THP-1-KO cells were generated using the CRISPR/Cas9 system. A plasmid containing mCherry Cas9 and a U6 promoter-driven guideRNA against STING, MAVS or cGAS was electroporated into THP-1 cells under the following conditions: 5 μg plasmid were mixed with 250 μl cell suspension (10 × 10^6^ ml^−1^ in OptiMEM) and electroporated at 950 μF and 250 V. Cells were FACS sorted for mCherry expression 24 h after electroporation. Positive cells were diluted under limiting conditions and plated in 96-well plates to obtain single-cell clones. The genotype of THP-1 clones was analysed by deep sequencing (Illumina, MiSeq).

### RT–qPCR

For analysis by RT–qPCR RNA was isolated using the High pure RNA isolation kit (Roche) and Taqman primer for hCXCL10, hISG54, mCXCL10, mIFNb1 were purchased form Life Technologies.

### Cloning

For mSTING expression from lentiviral vectors, a lentiviral cloning vector was generated (pCCL-PGK-MCS-HA-IRES-eGFP) which can receive an mSTING cDNA PCR fragment into the multiple cloning site (MCS) placing the cDNA in reading frame with a 3′ hemagglutinin tag. For this, an intermediate vector was generated named pCCL-PGK-MCS, in which the eGFP sequence of pCCL-PGK-eGFP^32^ was replaced with a MCS containing AscI, XhoI and XbaI restriction sites. This was cloned using annealed oligos carrying BamH1 and XhoI-compatible overhangs (the latter designed to eliminate the XhoI site to render the XhoI site in the MCS unique): sense oligo: 5′-gatccaaaggcgcgccaaactcgagaaatctagaaaa-3′ and antisense oligo: 5′-tcgattttctagatttctcgagtttggcgcgcctttg-3′. For constructing pCCL-PGK-MCS-HA-IRES-eGFP, the hemagglutinin-tag followed by IRES-eGFP was PCR-amplified from a retroviral transfer vector carrying mSTING (MSCV2.2-mSTING-IRES-eGFP; a kind gift from Russell Vance) using the following primers: forward: 5′-aaactcgagtatccttatgacgtgcctgactatgcc-3′, reverse: 5′-aaatctagagccgctttacttgtacagctcgtccatg-3′ and cloned into pCCL-PGK-MCS using the XhoI and XbaI sites. mSTING_R168A_ was generated using overlap extension PCR with the internal primers carrying the mutation: forward: 5′-tacattgggtacttggccttgatcttaccagggctc-3′ and reverse 5′-ggccaagtacccaatgtagtatg-3′ As a negative-control vector, pCCL-PGK-eGFP^32^ was used. Expression vectors for hSTING in HEK293T cells were constructed as described previously^22^. In brief, Mouse Sting was cloned into pcDNA3 with a carboxy terminal hemagglutinin tag. Mutations in Sting were generated using the QuikChange Site-directed mutagenesis kit (Stratagene) according to the manufacturer's guidelines. hSTING_140−379_ (CTD) containing an Arg at position 232, was PCR-amplified and cloned into pET28a for bacterial expression.

### Production of lentiviral vectors

Lentiviral vectors were produced in HEK293T cells seeded the day before transfection in 15-cm dishes at 10^7^ cells per dish. Cells were transfected using standard calcium phosphate co-transfection of the three lentiviral packaging plasmids (pRSV-Rev: 7.26 μg, pMD2.G: 9.07 μg and pMDLg/pRRE) and 31.46 μg of the lentiviral transfer vector. One day after transfection, medium was changed and 2 days after transfection, viral supernatant was filtered through a 0.45-μm filter and then ultracentrifuged through a 20% sucrose cushion at 25,000 r.p.m. at 4 °C for 2 h followed by resuspension of the pelleted virus in PBS. The yield of each vector production was determined by p24 ELISA (XpressBio, Thurmont, MD, USA) following the manufacturer's protocol.

### Protein purification

The pOAS1 protein was expressed in *E. coli* and purified by immobilized metal ion affinity chromatography followed by SEC as previously described^33^. hSTING 140–379 was expressed and purified by immobilized metal ion affinity chromatography in the same manner as pOAS1 and afterwards applied to a HiLoad16/60 Superdex 75 column (GE Healthcare). The protein was eluted with SEC buffer (20 mM Tris, 150 mM NaCl, 5% (v/v) glycerol (pH 7.5)) and fractions containing the protein were pooled and then concentrated to a concentration of 3.2 mg ml^−1^ using an Amicon Ultra centrifugal filter device.

### Analytical SEC

The peptide was first dissolved in 8 M urea and then mixed with STING 140–379 or pOAS1 in (20 mM Tris, 150 mM NaCl, 5% (v/v) glycerol (pH 8.5)) and incubated for 30 min at room temperature. The final concentrations were 66 μM for the peptide, 20 μM for the proteins and 0.8 M for urea. The peptide/proteins were then applied to a Superose 12 10/300 GL column (GE Healthcare) and eluted with SEC buffer (20 mM Tris, 150 mM NaCl, 5% (v/v) glycerol (pH 8.5)).

### Protein assays and confocal microscopy

Cellular lysates for detection of STING dimerization by western blotting were prepared using non-reducing RIPA lysis buffer with 0.2% SDS and complete mini inhibitor (Sigma). For detection of pTBK1 NaF (10 mM) was added to the lysis buffer. Separation by SDS-page was perfomed using Criterion precast gels (Biorad) and the protein transfer by Biorad Trans-blot turbo system. XT Sample buffer and reducing agent was also purchased from Biorad. For STING dimerization blotting of STING was performed using a mRabbit antibody (D2P2F, dilution 1:1,000) from Cell Signaling Technology. Antibodies for pTBK1 was purchased also from Cell Signaling Technology (dilution 1:1,000). hemagglutinin (dilution 1:1,000) and Giantin (dilution 1:1,000) antibodies were purchased from Abcam. Secondary antibodies for confocal imaging and western blotting were purchased from Invitrogen (dilution 1:500) and Jackson ImmunoResearch (dilution 1:10,000), respectively. Uncropped immunoblots are shown in Supplementary Fig. 9. For confocal imaging cells were permeabilized with ice-cold methanol and then blocked in 1% bovine serum albumin. Cells were then stained with respective antibodies, with DAPI for nuclear staining, mounted in proLong Gold (Molecular probes) and vizualised using a Zeiss LSM 710 laser-scanning microscope.

### Measurements of interferon-α/-β bioactivity

Murine interferon-α/-β was measured as described previously^34^. In brief, L929 cells were plated and cultured overnight in the presence of the samples for analysis in serial dilutions. L929 cells were then infected with VSV and incubated for another 2 days. interferon levels were then evaluated by the extend of virus induced cell death compared with interferonalpha controls. Human interferon-α/-β was measured as described by others^29^.

### Statistics

For analysis of statistically significant difference between two groups of data we used two-tailed Student's *t*-test when the data exhibited normal distribution, and Wilcoxon rank-sum test when the data set did not pass the normal distribution test.

## Additional information

**How to cite this article:** Holm, C. K. *et al.* Influenza A virus targets a cGAS-independent STING pathway that controls enveloped RNA viruses. *Nat. Commun.* 7:10680 doi: 10.1038/ncomms10680 (2016).

## Supplementary Material

Supplementary Information Supplementary Figures 1-9
