# Supplementary material for: Influenza A virus targets a cGAS-independent STING pathway that controls enveloped RNA viruses
Source: Nat Commun. 2016 Feb 19;7:10680. doi: 10.1038/ncomms10680 (PMC4762884; doi:10.1038/ncomms10680)
Supplement: Supplementary Information — Supplementary Figures 1-9 [file ncomms10680-s1.pdf]

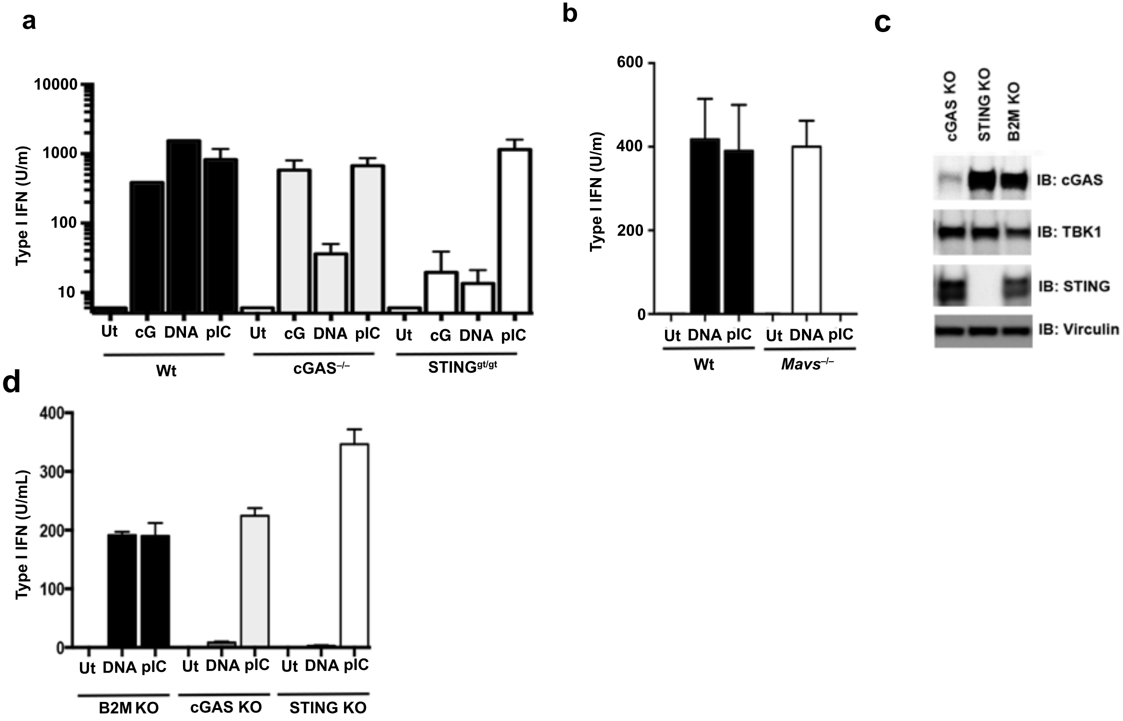

Supplementary Figure 1.
(**a+b**) MEFs from either wt, cGAS$^{-/-}$, Sting$^{gt/gt}$, and MAVS$^{-/-}$ were tested for ability to react with cGAMP (cG), DNA, and poly I:C (pIC). After 16hrs of stimulation supernatants were analyzed for type I IFN by bioassay. Each bar represents mean and SEM from four biological samples. (**c**) Knockout of cGAS, STING and B2M (as a control) in THP-1 cells was performed by the CRISPR/Cas9 system. Clones were then analyzed for expression of target proteins by western blotting. Vinculin was used as loading control. (**d**) THP-1 clones were tested for functional KO by stimulation with DNA and poly I:C (pIC) and subsequent analysis for type I IFN produciton.

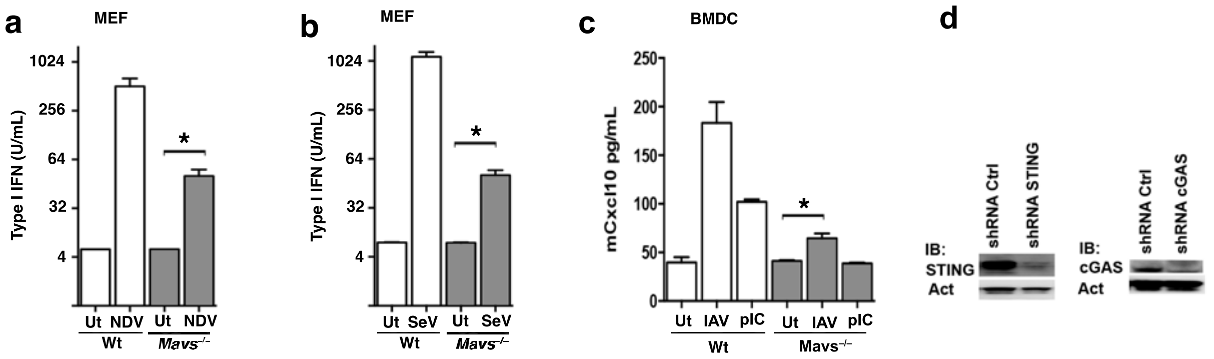

Supplementary Figure 2.
Primary mouse embryonal fibroblast from wt and Mavs deficient mice were infected with either NDV (**a**) or SeV (**b**). IFN levels in the supernatant was measured after 20 hours of infection. (**c**) BMDCs from either wt or MAVS deficient mice were infected with IAV (moi 15) for 20 hours. Supernatants were analyzed for the ISG CXCL10 by ELISA. Graphs represent one of two independent experiments with bars indicating mean +sem of at least four biological samples. ”*” indicates significant difference evaluated by students t test.(**d**) Ctrl, STING, or cGAS targeting shRNA was expressed by lentiviral delivery in THP-1 cells deficient in MAVS. Knockdown was evaluated by WB as indicated in figure.

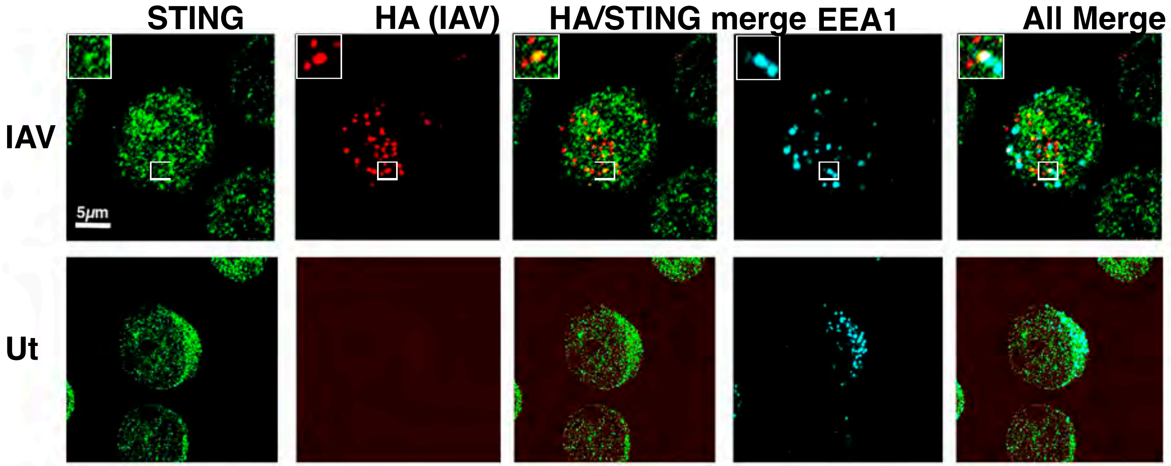

Supplementary Figure 3: THP-1 celles were infectied with IAV (PR8 strain) for 1 hour. Cells were thenfixed and stained for STING (green), the IAV surface antigen Haemagluttinin (HA, red), and the early endosome marker A1 (EEA1, cyan). Cells were then analyzed by confocal microscopy.

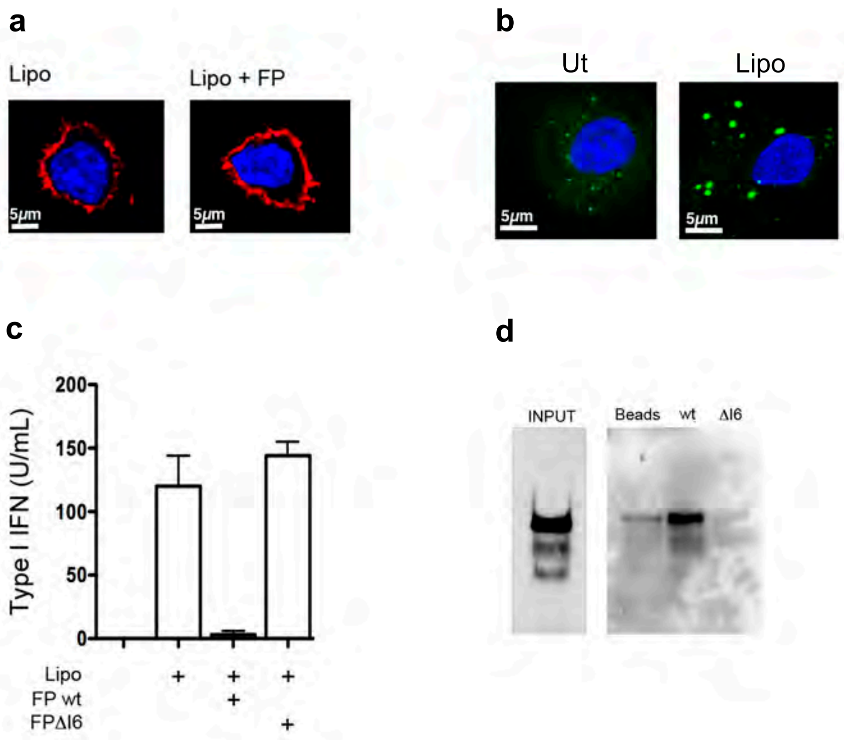

Supplementary Figure 4.
(**a**)THP-1 cells were stimulated with fusogenic liposomes (Lipo) that stain red due to the content of lissamine rhodamine labeled lipids (red) for 10 minutes. Before liposome stimulation cells either pretreated with IAV fusion peptide (FP) or no pretreatment. Cells were then fixed and stained with DAPI before analysis by confocal microscopy. (**b**) Human monocyte derived macrophages (hMDMs) were either left untreated (left) or stimulated with fusogenic liposomes (right) for 4 hours. Cells were then fixed, stained with the nuclear stain DAPI (blue) or with anitbodies against STING (green). Cells were analyzed using laser scanning microscopy. (**c**) BMDCs were either left untreated or stimulated with fusogenic liposomes. Before stimulation cells were treated with FPwt or the FP variant FPΔI6. (**d**) Western blot analysis of precipitates from pulldowns using either biotinylated FPwt or biotinylated FPΔI6.

a

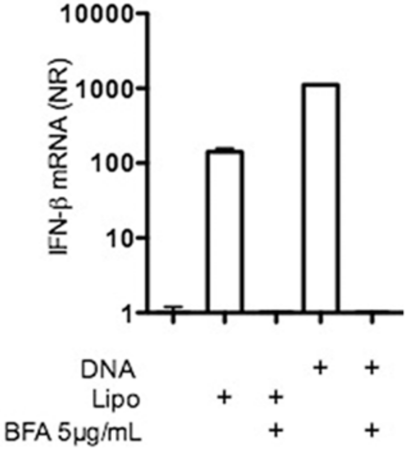

Supplementary Figure 5.

(**a**) BMDCs were pretreated with the golgi transport inhibitor Brefeldin A (BFA 5μg/mL) for 30 minutes. Cells were then treated with fusogenic liposomes (Lipo) or transfected with immuno-stimulatory DNA (DNA, 4μg/mL). After 5 hours of stimulation mRNA was collected and analyzed for Ifnβ mRNA (normalized to beta-actin mRNA contents). All experiments were performed at least two times with similar results. Bars and error bars indicate mean and SEM.

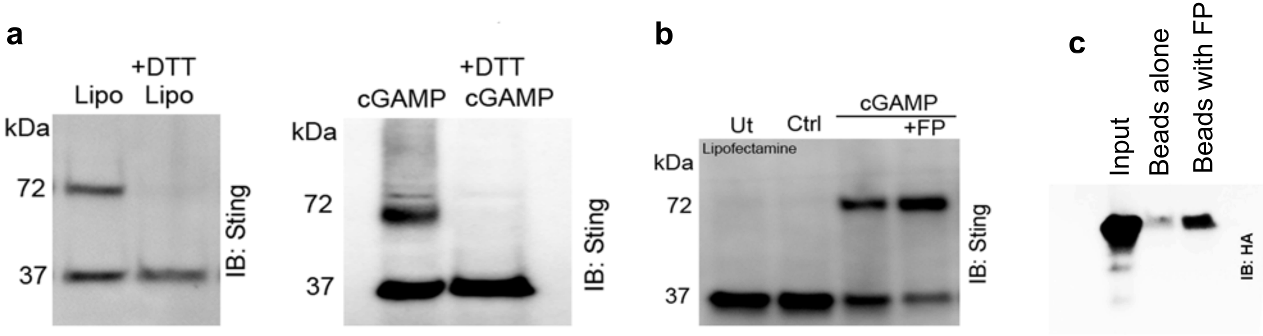

Supplementary Figure 6.
(**a**) Lysates from THP-1 cells stimulated with either fusogenic liposomes (Lipo) or with cGAMP (10nM, digitonin delivery) for 3.5 hours was either kept at non-reducing conditions or treated with the reducing agent DTT. Lysates were then assayed by SDS-PAGE and probing for STING.
(**b**) THP-1 cells were transfected with 12μM cGAMPusing Lipofectamine2000. After 2.5 hours cells were harvested and lysed in non-reducing lysis buffer and separated using nonreducing SDS-PAGE. STING was then visualized by western blotting.
(**c**) HEK293 cells expressing HA-tagged STING were lysed and subjected to pull-down analysis using either streptavidin beads alone or streptavidin beads incubated with biotin-tagged FP. Pull-downs then analyzed by westerb blotting for HA.

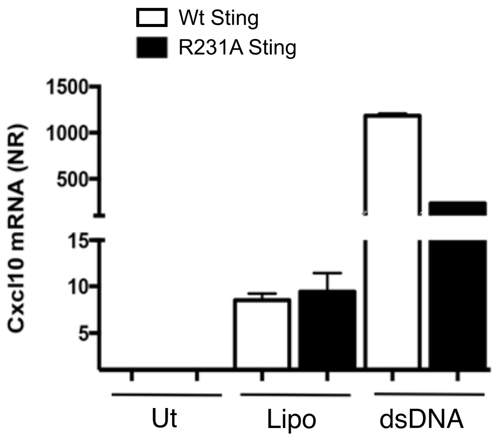

Supplementary Figure 7. Wt mSTING or R231A mSTING was stably expressed in BMDCs from Tmem173gt/gt mice. Cells were either left untreated or stimulated for 4 hours with fusogenic liposomes (Lipo) or by transfection with immunostimulatory dsDNA (dsDNA). Samples were then analyzed for Cxcl10 mRNA by qPCR. Bars depict mean +sem of two biological samples.

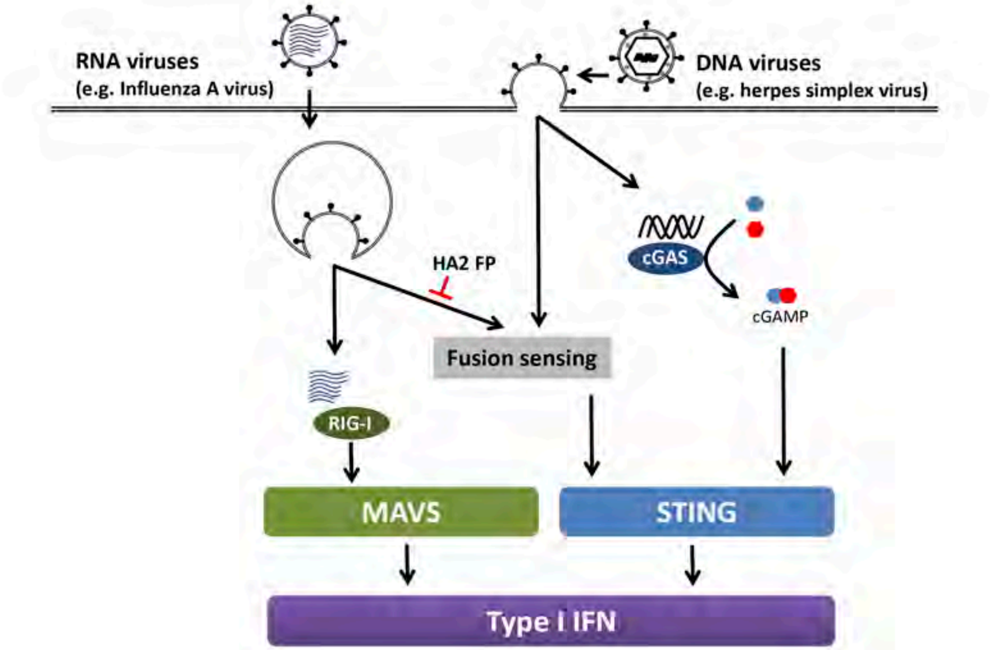

Supplementary Figure 8.
Model for how the influenza A virus fusion peptide of hemagglutinin selectively interferes with the fusion-activated STING-dependent signaling pathway.

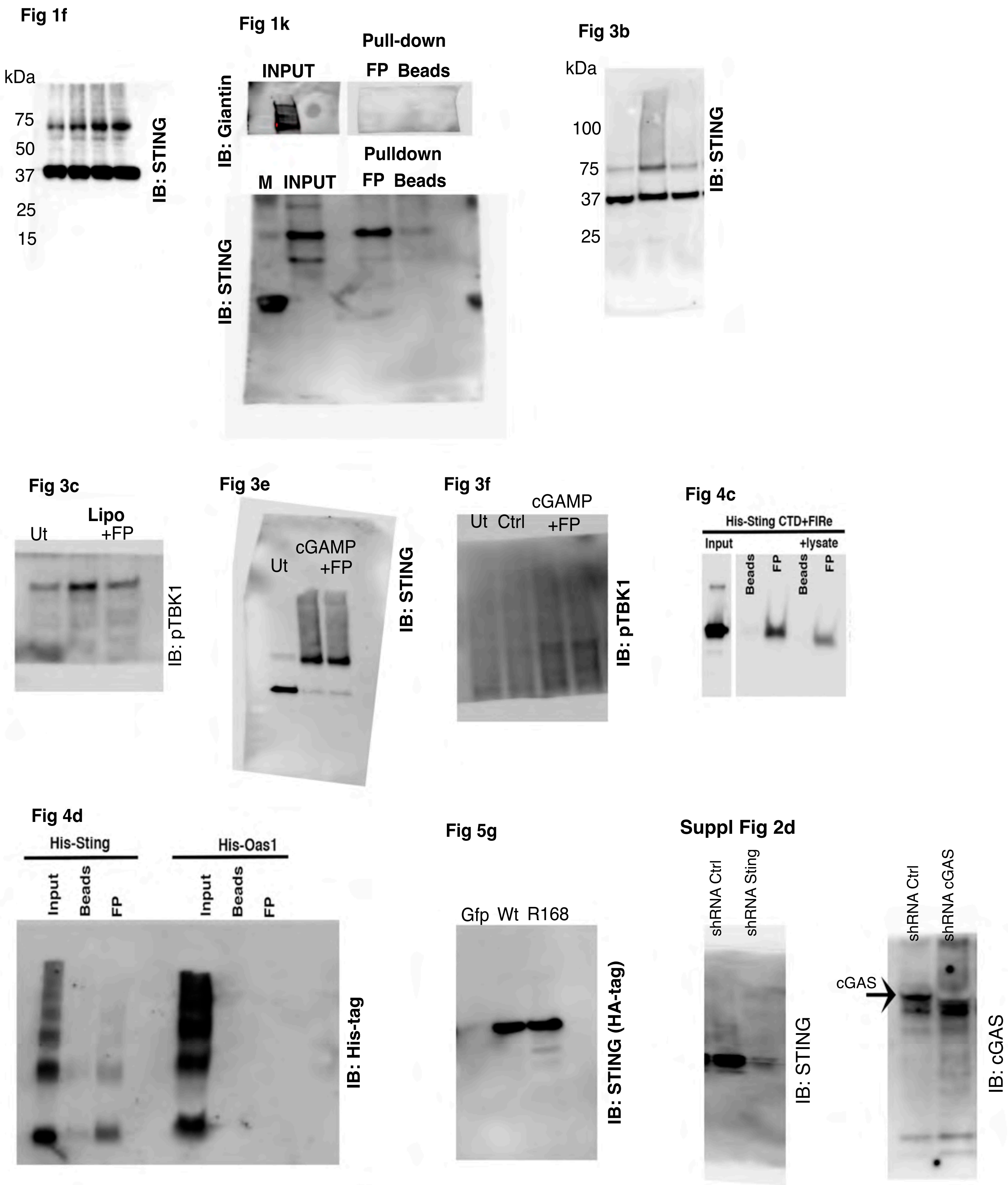

Supplementary Figure 9.
Raw uncropped blots from selected figures as indicated in each panel.
